# Conflict and adaptation signals in the anterior cingulate cortex and ventral tegmental area

**DOI:** 10.1038/s41598-018-30203-4

**Published:** 2018-08-06

**Authors:** Thomas W. Elston, Shivam Kalhan, David K. Bilkey

**Affiliations:** 1 Department of Psychology, University of Otago, Dunedin, 9016 New Zealand; 2 Brain Health Research Centre, University of Otago, Dunedin, 9016 New Zealand; 3 Present Address: Institute for Neurobiology, University of Tübingen, Tübingen, 72076 Germany

## Abstract

The integration and utilization of feedback in order to determine which decision strategy to use in different contexts is the core of executive function. The anterior cingulate cortex (ACC) is central to these processes but how feedback is made available to the ACC is unclear. To address this question, we trained rats with implants in the ACC and the ventral tegmental area (VTA), a dopaminergic brain region implicated in feedback processing, in a spatial decision reversal task with rule switching occurring approximately every 12 trials. Following a rule switch, the rats had to shift and sustain responses to the alternative side in order to obtain reward. Partial directed coherence (PDC) models of signal directionality between the ACC and VTA indicated that VTA → ACC communication (near 4 Hz) increased immediately prior to incorrect choices and during post-error decisions. This increase did not occur during correct choices. These data indicate that the VTA provides a feedback-driven, bottom-up modulating signal to the ACC which may be involved in assessing, and correcting for, decision conflict.

## Introduction

The anterior cingulate cortex (ACC) appears central to feedback utilisation and adaptive decision-making as damage to, or inactivation of, the structure impairs an organism’s ability to integrate recent outcome information so as to adjust and optimize goal pursuit^1–4^. The ACCs role in these processes has been conceived as involving ensembles of ACC neurons that dynamically encode the task at hand such that modulation of these neuronal task models drive behavioural change^5–7^. In support of this model, subpopulations of rodent ACC neurons appear to encode the goal values and actions associated with reward acquisition optimisation^8^. Furthermore, recordings of monkey ACC neurons indicate that the ACC tracks environmental change^7^ and several previous studies have shown that neural activity in the ACC is modulated by feedback and that feedback-linked responses strongly predict subsequent behavioural change^7,9–13^. This feedback activity is thought to underlie the integration of new information into existing task representations as a means of adapting future behaviour to a changing environment^12–15^. The connectivity underlying this feedback mechanism is, however, still unclear.

One possibility, suggested by computational models^16,17^ and studies in rodents^18,19^, monkeys^3,20^, and humans^21^ is that mesocortical dopaminergic (DA) innervation of the prefrontal cortex (PFC; including the ACC) from the ventral tegmental area (VTA) could convey feedback which could then modify ACC neuronal representations. For instance, Ellwood *et al*.^19^ found that photostimulation of DA terminals originating in VTA and terminating in PFC altered choice behaviour, with tonic, low-frequency (~5 Hz) stimulation increasing the likelihood that mice would persist in a prior behaviour, while phasic, high-frequency (~50 Hz) photostimulation increased the likelihood of a switch in behaviour, regardless of the prior (rewarded or not) outcome. Consistent with this finding, blockade of DA D2 receptors has been shown to reduce the ability of rodents to flexibly switch between rule-based response-strategies^22^ and increases in ACC DA concentrations have been detected as humans complete a rule-based card sorting task^23^. Furthermore, DAergic stimulation/inhibition differentially influences the coding responses of individual rule-selective PFC neurons^24,25^. Together, these data suggest that VTA-to-ACC signalling might influence the firing properties of ACC neurons that represent the task at hand so as to modify task models and thereby initiate behavioural flexibility on the basis of feedback from the environment. What is missing in current conceptions of this system is, however, information about how the electrophysiological relationship between the ACC and VTA changes during this process and how this reflects communication between the two regions.

**Figure 1:**
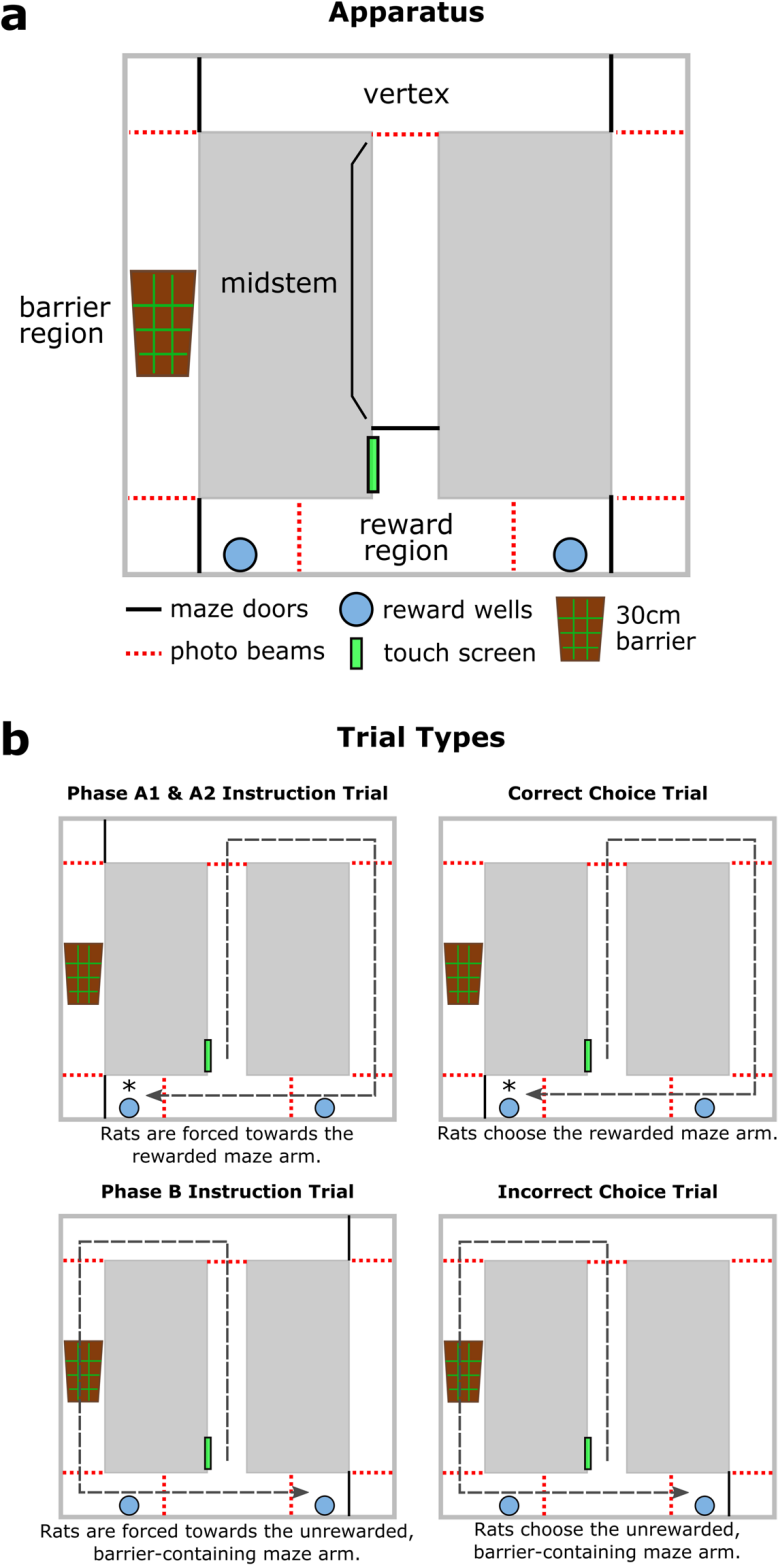
Apparatus (**a**) and task trial-type diagrams (**b**). In (**b**), the asterisk (*) indicates the rewarded well. We trained the rats to run all the way through the reward zone to the far reward well so that they were well away from the photobeam-triggered gate which was raised at the completion of each trial.

To examine the role of VTA-to-ACC signalling during feedback-driven, adaptive decision making, we simultaneously monitored ACC and VTA LFPs of rats performing a spatial decision reversal task (see Fig. 1). In this task, rats had to decide whether to turn left or right in a maze such that in any block of trials only one choice yielded reinforcement. Every 12 trials (approximately), the rule switched and the rats needed to shift and sustain responses to the alternative side to obtain reward. We examined task-dependent changes in LFP power and coherence and assessed signal directionality between the ACC and VTA through the use of partial directed coherence (PDC) modelling^26,27^. On the basis of prior studies indicating that the ACC and VTA communicate via slow (~4 Hz) oscillations^28–30^ and that such oscillations in the cortex are modulated by DA^18,31^, we hypothesised that 4 Hz VTA-to-ACC PDC model magnitudes would be maximal as an animal responded to error signals from the environment and subsequently adapted its behaviour.

## Results

### Task, apparatus, and neurophysiological recordings

**Figure 2:**
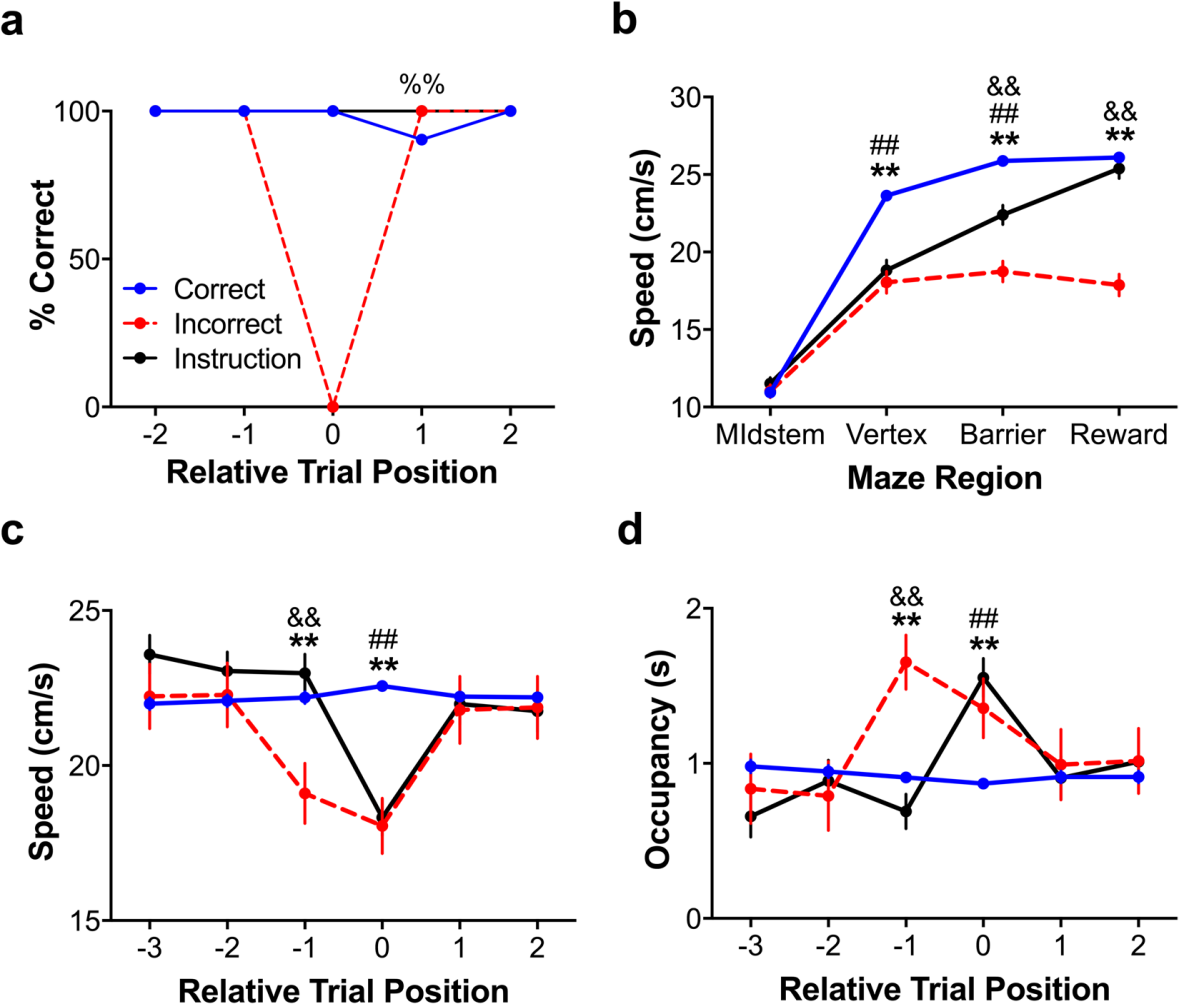
Maze region and trial type dependent changes in behaviour. See also Fig. S1. (**a**) Percent correct (reinforced) decisions relative to each trial type. Animals never repeated an error, indicating post-error adaptation. (**b**) Linearized running speeds by trial type and maze region. The rats ran fastest through the vertex region when making a correct choice and were slowest in implementing their decision during incorrect choice trials. The running speed gathered from the more conservative definition of the vertex region, as described in the main text, is presented here. (**c**) Vertex running speeds relative to each trial type. During post-error trials, animals’ running speeds were not different from trials following a correct choice, indicating that the animals adapted their decision behaviour following an error. Rats ran slower on the trial immediately prior to an error trial, perhaps because they ran back and forth through the vertex region in uncertainty. (**d**) Vertex occupancy relative to each trial type. Animals spent more time in the vertex during incorrect and forced trials as well as during the putative ‘lookback’ events during trials immediately prior to making an incorrect (unrewarded) choice. These data from phase A1 are presented as mean ± SEM although they were analyzed as residuals (see Methods). ^%%^Indicates p < 0.005 via Fisher’s Exact Test; ^**^Indicates that correct choices were significantly different from incorrect choices at the p < 0.005 significance level via multiple comparisons tests; ^&&^Indicates that incorrect and instruction trials were significantly different at the p < 0.005 significance level via multiple comparisons tests; ^##^Indicates that correct choices and instruction trials were significantly different at the p < 0.005 significance level via multiple comparisons tests.

The aim of these experiments was was to determine whether and how and ACC and VTA interact during feedback-driven adaptive decision making. In particular, we were interested in whether the rats’ behaviour and underlying neural activity would differ when comparing responses during and following feedback in the form of errors or instructions and whether the behaviour and neural activities attending these feedback events would differ from that on correct choice trials. To assess these questions directly, we simultaneously monitored the ACC and VTA LFPs of five rats trained to perform a spatial-decision, reversal task (see Figs 1 and 2). Each recording session was comprised of six blocks of 12 ± 2 trials such that the “correct” (rewarded and non-effortful) and “incorrect” (unrewarded and effortful) maze arms reversed on each block. Each block began with an “instruction” trial which, depending on the phase of the experiment (see Methods), forced the animals towards either the “correct” or “incorrect” maze arm. The remaining trials in a block were free choice trials (see Methods). The inclusion of these “instruction” trials allowed us to assess whether and how instructional (non-volitional) and error-related (volitional) feedback differentially influenced behaviour and ACC-VTA neural activities. Of the 998 trials (across all 5 rats) included in our phase A1 analyses, 835 were correct choices, 73 were incorrect choices, and 90 were instruction trials. Because of the relatively few errors made by each rat, we adapted Ma *et al*.’s^32^ regression-based approach to controlling for between-subject variance (see Methods). This approach allowed us to pool individual trials across animals so that we could analyze them via multifactorial ANOVAs.

### Dynamics of decision behaviour

Because we were interested in the impact of instructional and error-related feedback on decision behaviour, we examined the distribution of the rats’ choices. The rats never repeated an error within two trials of an error or instruction trial (Fig. 2a) and they were significantly more likely to generate an error on the trial immediately after a correct trial than immediately after an error trial (Fisher’s Exact Test, p = 0.0018). These results indicate that both types of feedback (instructional and error-related) influenced the rats’ future decision behaviour.

Because the decision patterns associated with the different types of feedback (instructional and error-related) were similar, we wondered whether the overt behaviours of the animals would also be similar. To assess this, we obtained the mean running speed (see Methods) for each maze region for each lap for each animal, separately (see Fig. 2b). We then regressed off the between-subject variance (see Methods) and used the ANOVA to determine how running speed varied with the factors of maze region (midstem, vertex, barrier, reward) and trial type (instruction, correct, incorrect). We found no significant main effects of trial type or maze region but did find a highly significant trial type x maze region interaction (F_1,4047_ = 33, p < 0.0005).

This interaction effect reflects (i) that the rats’ ran slower in the vertex during incorrect choices and instruction trials than during correct choices, respectively (both p < 0.0005, multiple comparisons test; *multcompare* in MATLAB); (ii) that rats were slowest running through the barrier region during incorrect choice trials and fastest during correct choice trials (p < 0.005, multiple comparisons tests), likely due to the time required to climb the barrier; and (iii) that the rats were slowest moving through the reward region during incorrect choice trials as compared to both instruction and correct choice trials (p < 0.005, multiple comparisons tests). The slower running speed during incorrect choice trials may reflect the animal being less motivated, as those trials were both effortful and unrewarded. In contrast, because, in phase A1, the correct choice and instruction trials were rewarded, the elevated running speed may indicate the animals anticipated the reward and were therefore more motivated to attain it.

Because we found that the rats ran through the vertex at different speeds during different trial types, we wondered whether their movement patterns at the choice point would also be different. When we plotted the animals’ tracked positions for each trial type (see Fig. S1 for example single trials), we did not see any notable differences in the vertex-region trajectories of the rats during correct and incorrect choices. It was also clear that the reduced running speed during instruction trials was the result of the animal tending to initially run towards the default (prior correct) choice. From examining the trajectories of incorrect trials, in particular, it is clear that the animals did not stop moving in the vertex, they just moved more slowly.

Because we found differences in the overt behaviours associated with the different types of feedback events (instructional and error-related), we next asked if there were differences in overt behaviour during post-feedback adaption. We did this by conducting an ANOVA considering the effects of relative trial position (i.e. trials immediately before the trials of interest, the trials of interest, and the trials subsequent to the trials of interest) and trial type on running speed. Although we did not detect significant main effects of trial type or trial position, there were significant trial type x trial position interactions (F_1,5993_ > 14, p < 0.0005). The animals’ vertex running speed on trials following an error were indistinguishable from running speeds on trials following correct choice and instruction trials (p > 0.1, multiple comparisons test). These results suggest that animals were not prone to perseveration errors, possibly because they integrated outcome-related feedback during incorrect and instruction trials and adapted their decision making accordingly. Interestingly, the rats ran slower during the trial immediately prior to an error as compared to the trials immediately preceding a correct choice or instruction trial, respectively (see position −1 in Fig. 2c; both p < 0.005, multiple comparisons test). Such reduced movement speeds at the choice point during incorrect choices is consistent with data from numerous studies demonstrating that greater choice reaction times correspond to greater choice conflict^12,33,34^. Thus, such increased decision times may indicate that the animals experienced choice conflict when making decisions which, ultimately, were incorrect.

### Task-related changes in 1–30 Hz LFP power and coherence

**Figure 3:**
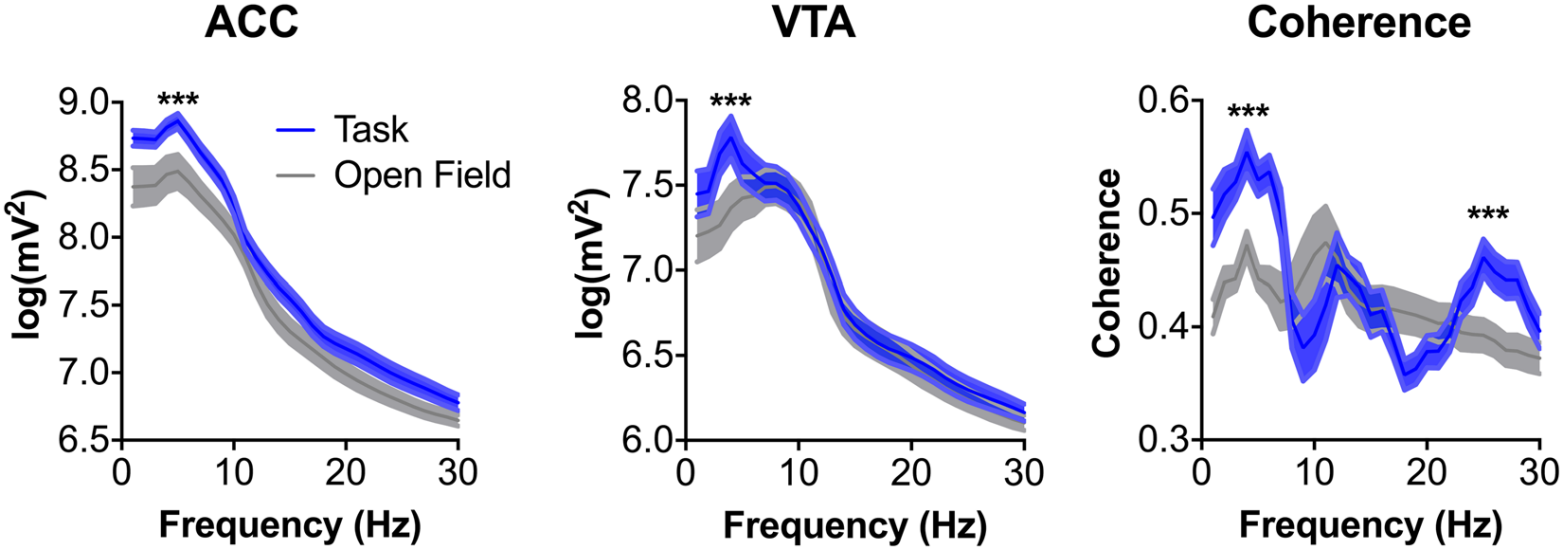
ACC and VTA power and coherence in the 3–5 Hz band were significantly greater during the task as compared to recordings taken during open field recordings collected immediately before or after each data collection session. Although comparisons were computed for each animal separately (n = 9 paired open field and task recordings per animal), the data here are presented as the grand mean ± SEM across all animals (n = 5 animals).

Because the ACC and VTA are implicated in decision conflict and feedback processing^3,20,35,36^, we asked whether the electrophysiological activity of, and interactions between, the ACC and VTA differed during the different types of feedback and adaptation. Our first step was to determine whether the task engaged the simultaneously recorded ACC and VTA LFPs (see Fig. S2 for histology). We did this by computing the ACC and VTA LFP power spectrum density (PSD) functions in the 1–30 Hz band. These were characteristically different in the maze with the ACC having a primary peak near 4 Hz whereas the VTA exhibited local peaks at 4 Hz and 8 Hz (see Fig. 3). The ACC and VTA were maximally coherent in the 3–5 Hz frequency band. As a comparison, we also computed the PSDs and coherence for recordings of the animals roaming in an open field which were paired with each animals’ recording sessions. Although the PSDs across the 1–30 Hz band tended to be greater during the maze task as compared to the open field recordings, only ACC-VTA coherence in the 3–5 Hz band was significantly elevated during the task as compared to in the open field (p < 0.005 for each animal, paired t-tests, n = 9 sessions per animal). Because we had found in our previous study of the ACC-VTA circuit^28^ an effect across a wide 4–12 Hz band, we were surprised to not detect a consistent significant difference there. However, we note that the maximal portion of the PSDs computed for that study were at 4 Hz, consistent with our findings here and recent work by others^18,31^. We also found a task-related elevation in beta (20–30 Hz) power (p < 0.005 for each animal, paired t-tests, n = 9 sessions per animal). However, because several other studies of the mPFC (including the ACC) have reported a functionally relevant 3–5 Hz oscillation^29,30,37,38^ and to retain focus in this paper, we focused subsequent analyses on the 3–5 Hz band, hence referred to as 4 Hz.

### Conflict-related changes in ACC and VTA 4 Hz power and coherence

**Figure 4:**
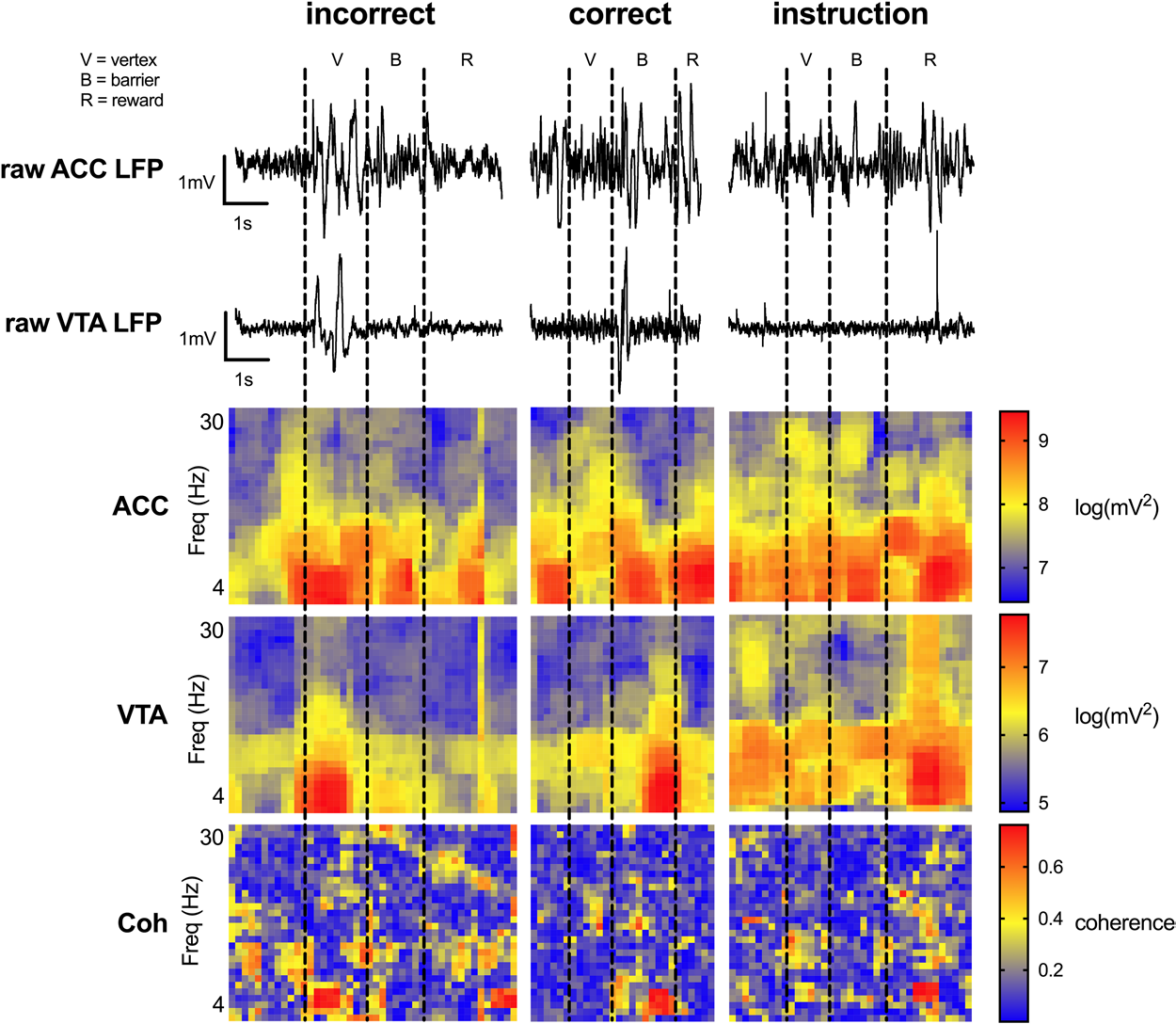
Single trial examples of raw ACC and VTA LFP traces and the spectrograms generated from them for each trial type. These data are from one recording session in rat 4 during phase A1.

Having established that the ACC and VTA LFPs were modulated at ~4 Hz, we asked whether/how 4 Hz activities varied with respect to the different aspects of our task. We did this computing the PSDs and coherence for each maze region, for each trial type, for each trial, for each rat, separately. In order to pool trials across animals, we first normalised each animals’ trial-by-trial data with respect to the variance across the entire maze via the feature scaling formula^39^. We then used Ma *et al*.’s^32^ regression approach to control for between-subject variance (see Methods) and conducted all subsequent analyses on the residuals unless specifically noted. This procedure revealed that the factor of Subject accounted for a small proportion of the variance for each electrophysiological measure (R^2^_ACC_ = 0.008; R^2^_VTA_ = 0.037; R^2^_Coh_ = 0.002; R^2^_VTA-to-ACC PDC_ = 2.390 × 10^−4^). See Figs S3–S5 for data processed without this regression procedure. See Fig. 4 for raw LFP traces and the spectrograms generated from them during single trials of each trial type.

**Figure 5:**
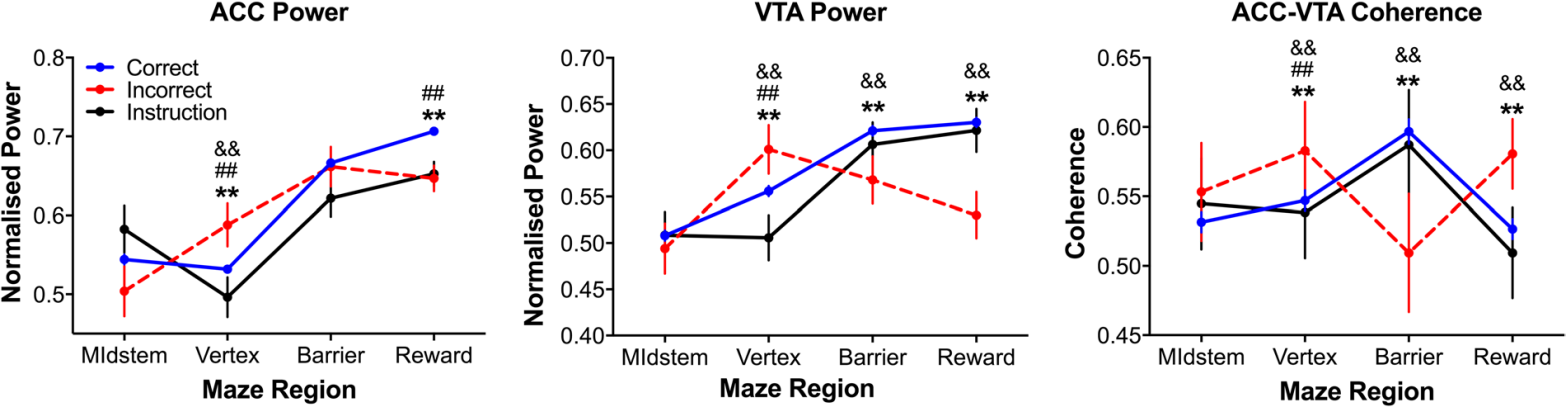
Maze region and trial type dependent changes in 4 Hz power and coherence. 4 Hz ACC-VTA coherence was significantly greater in the reward zone during incorrect trials, as compared to other trial types. These data from phase A1 are presented as mean ± SEM. ^**^Indicates that correct choices were significantly different from incorrect choices at the p < 0.005 significance level via multiple comparisons tests; && indicates that incorrect and instruction trials were significantly different at the p < 0.005 significance level via multiple comparisons tests; ^##^Indicates that correct choices and instruction trials were significantly different at the p < 0.005 significance level via multiple comparisons tests. See Fig. S3 for data processed without the regression procedure.

ACC and VTA 4 Hz power and coherence in the vertex were elevated during incorrect choices as compared to both correct choice and instruction trials (see Fig. 5; ANOVAs: main effect of region: none significant; main effect of trial type: none significant; region x trial-type interaction: all F_6,4047_ > 5, all p < 0.0005; all p < 0.005, multiple comparisons tests; see Fig. S3 for data processed without the regression procdure). These results indicate that ACC and VTA power and coherence were maximal as the animals carried out incorrect choices. To the extent that the increased choice time associated with incorrect choice trials are indicative of choice conflict, these results suggest that ACC and VTA signal magnitudes vary with the degree of decision conflict experienced by the animal such that greater decision conflict corresponds to greater ACC and VTA 4 Hz power and coherence. Such conflict-related findings for the ACC, particularly, have been reported previously^12,34,40,41^.

ACC 4 Hz power in the barrier region did not vary significantly across the trial conditions (all p > 0.05, multiple comparisons tests); however, VTA 4 Hz power and ACC-VTA 4 Hz coherence were significantly reduced in this region during incorrect choice trials as compared to both correct choice and instruction trials (p < 0.005, multiple comparisons tests). Reward region ACC 4 Hz power was significantly greater during correct choice trials when compared to both incorrect choice and instruction trials (p < 0.005, multiple comparisons tests). Reward region VTA 4 Hz power during incorrect choices was significantly less than during correct choice and instruction trials (p < 0.005, multiple comparisons tests); however, no differences between reward region VTA 4 Hz power during correct choices and instruction trials were detected. Interestingly, reward region ACC-VTA 4 Hz coherence during an incorrect choice trial was greater than during correct choice and instruction trials (p < 0.005, multiple comparisons tests). These results indicate communication between the ACC and VTA increased when the outcome was unrewarded.

### Adaptation-related changes in ACC and VTA 4 Hz power and coherence

**Figure 6:**
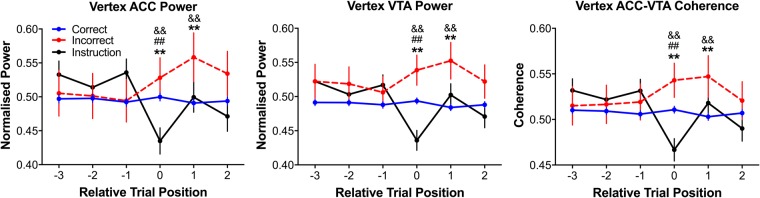
Changes relative to trial types of interest in ACC and VTA 4 Hz power and coherence. Trial position 0 in these plots indicates the normalised residual vertex mean of all trials of each type for each dependent measure, respectively. The data represented at trial position 1 are the vertex means of all trials immediately after each trial that was used in trial position 0. Other trial position data were calculated in a similar fashion. Consistent with the linearized results, vertex ACC and VTA 4 Hz power and coherence were greatest during incorrect choice trials as compared to during instruction and correct choice trials. ACC and VTA 4 Hz power and coherence were elevated in the vertex region as the animal made a post-error correct choice. These data from phase A1 are presented as mean ± SEM of all normalised residual data where the normalisation was relative to the vertex. ^**^Indicates that correct choices were significantly different from incorrect choices at the p < 0.005 significance level via multiple comparisons tests; && indicates that incorrect and instruction trials were significantly different at the p < 0.005 significance level via multiple comparisons tests; ^##^Indicates that correct choices and instruction trials were significantly different at the p < 0.005 significance level via multiple comparisons tests. See also Fig. S4 for data processed without the regression procedure.

Because the behavioural and electrophysiological effects we observed in the vertex can be interpreted as relating to decision conflict and because animals adapted their decision strategies to a correct response within one trial post-error (Fig. 3a), we were interested in whether these 4 Hz signals would also be related to post-error adaptation. We assessed this by computing the ACC and VTA 4 Hz power and coherence in the vertex region for the three trials prior, and two trials after, a trial of each type (see Fig. 6). Because we were solely interested in the vertex region, we restricted our feature scaling normalisation procedure of the power and coherence measures to this region. That is, normalisation considered the variance in the dependent measures with respect to the variance in the vertex alone rather than the variance observed across the entire maze. Between-subject variance was then regressed off (see Methods) and the residual matrices were then analysed further.

We found no main effects of trial type or trial position in the three trials prior to the trials of interest (i.e. no differences between trial types across trial positions −3 to −1; see Fig. 6), likely because the trials occurring before each trial of interest were exclusively correct choice trials (see Fig. 3a). One-way ANOVAs considering only the ‘trials of interest’ (trial position 0 in Fig. 6) on the 4 Hz ACC and VTA power and coherence signals confirmed the earlier linearized analyses (Fig. 5) which suggested that conflict was likely minimal during forced, instruction trials, maximal during the extended decision time, incorrect choice trials, and normative during default, correct choice trials (all F_2,998_ > 40, all p < 0.0005; instruction <correct, correct <incorrect, instruction <incorrect, all p < 0.005, multiple comparisons tests).

Because we observed a clear behavioural adaptaion effect and that the ACC and VTA are implicated in adaptation, we asked whether the changes in vertex ACC and VTA 4 Hz power and coherence we observed during error and instruction trials were sustained across the following two trials (positions 1–2 in Fig. 6; see Fig. S4 for data processed without the regression procedure). Although we found no significant main effects of trial type and trial position, we did find significant trial type x trial position interaction effects for ACC and VTA 4 Hz power and coherence (all F_4,6175_ > 8, all p < 0.0005). Vertex ACC and VTA 4 Hz power and coherence in the trials immediately after instruction trials increased such that they were no different from trials following a correct choice (all p > 0.1, multiple comparisons test). In contrast, ACC and VTA 4 Hz power and coherence remained elevated on trials immediately following an incorrect choice as compared to the trials immediately after correct choices or instruction trials (all p < 0.005, multiple comparisons tests). No differences between the different trial types were detected by the second trial after a trial of interest (all p > 0.1, multiple comparisons tests). Together, these data indicate that communication between the ACC and VTA increased when the rats adapted their decisions following an error.

To further characterize these putative adaptation signals, we determined how the post-error increases in ACC and VTA signals compared with that occurring prior to the error trial. An ANOVA was run comparing the trials immediately before and after a trial of interest (trial positions −1 and 1 in Fig. 6). Although we found no main effects of trial type or trial position, we did find a significant trial type x trial position effect for each dependent measure, respectively (all F_2,1996_ > 7, all p < 0.0005). Consistent with our prior analyses, only post-error choices were significantly greater than prior-to-error choices (p < 0.005, multiple comparisons test). These results indicate that 4 Hz ACC-VTA communication was significantly greater following an error than before one.

### Task-modulated changes in LFP signal directionality

Our prior analyses indicated that ACC-VTA 4 Hz communication increased in the vertex during episodes of putative conflict and adaptation. However, the directionality of that communication was unclear. Therefore, to assess the directionality of communication between the ACC and VTA during these putative conflict and adaptation epochs, we implemented Boykin *et al*.’s^26^ method of partial directed coherence (PDC; see Methods). The mean PDC in the 3–5 Hz frequency band (hence 4 Hz) was calculated for each maze region, trial, and animal, separately. Because PDC is itself a normalised measure, we did not normalise the input data. The between-subject variance was accounted for according to the regression procedure described earlier (see Fig. S5 for analyses without the regression procedure). To verify the integrity of the PDC modelling, we scrambled the recorded signals from both ACC and VTA and conducted the trial-by-trial, region-by-region PDC analysis again. In the case of ACC → VTA directionality, the mean of the permutated PDC distribution was not significantly different from the non-permuted data (p > 0.05, t-tests, n = 1000 permutated PDC magnitudes vs 1000 non-permuted residual PDC magnitudes). In the case of VTA → ACC directionality, however, the mean of the permutated PDC distribution was significantly weaker than the non-permuted distribution (p < 0.0005, t-test), indicating that our VTA → ACC PDC results were significantly different from chance. We therefore focused subsequent analyses on the VTA → ACC PDC models.

**Figure 7:**
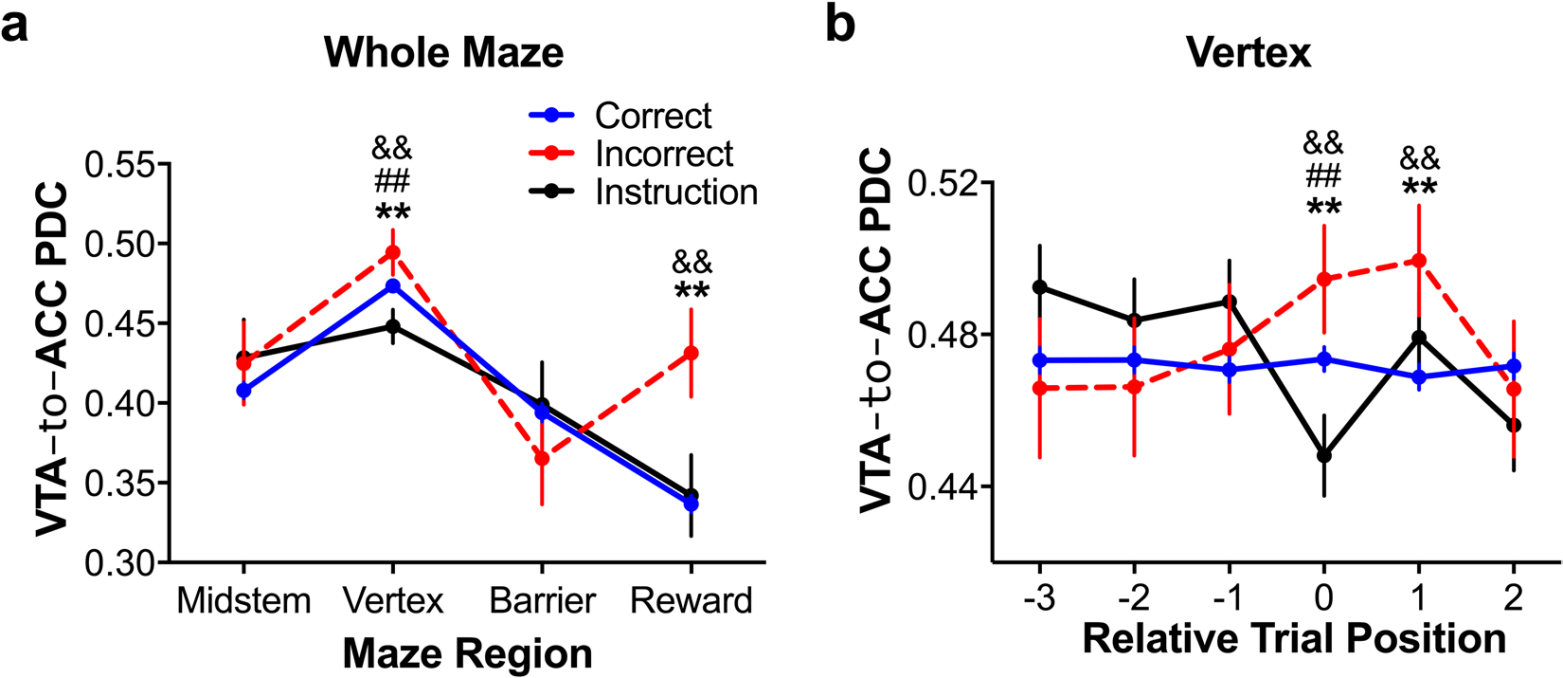
4 Hz VTA → ACC PDC by trial type and maze region (**a**) and by trial type and trial position in the vertex region (**b**). (**a**) VTA → ACC PDC was significantly greater in the vertex and reward regions during incorrect trials, as compared to the other trial types. (**b**) Vertex VTA → ACC PDC models were greatest during incorrect choice trials and the post-error trials immediately following. Vertex VTA → ACC PDC models were lowest in magnitude during instruction trials. No differences in VTA → ACC PDC models were detected during the three trials leading up to each trial of interest, respectively, and no effect of trial type was detected two trials post-trial of interest. These data from phase A1 are presented as the mean ± SEM of the residual PDC model magnitudes. Because PDC is itself a normalised measure, data were not normalised. ^**^Indicates that correct choices were significantly different from incorrect choices at the p < 0.005 significance level via multiple comparisons tests; && indicates that incorrect and instruction trials were significantly different at the p < 0.005 significance level via multiple comparisons tests; ^##^Indicates that correct choices and instruction trials were significantly different at the p < 0.005 significance level via multiple comparisons tests. See also Fig. S5 for data processed without the regression procedure.

In a result that was consistent with the 4 Hz coherence findings, VTA → ACC 4 Hz PDC significantly varied by maze region and trial type (Fig. 7a; region main effect: F_3,4047_ = 8.75, p < 0.005; trial type main effect: F_2,4047_ = 5.40, p < 0.005; trial type x region interaction: F_6,4047_ = 4.30, p < 0.005). This was because VTA → ACC PDC model magnitudes were, generally, greatest in the vertex and during incorrect choice trials. Post hoc multiple comparisons tests (both p < 0.005) confirmed that the interaction was a result of the VTA → ACC signal being significantly greater in the vertex and reward regions during incorrect choice trials. In another result that was consistent with the changes in vertex coherence, VTA → ACC PDC was greater during incorrect choices than during both correct choices and instruction trials and VTA → ACC PDC was greater during correct choices than during instruction trials (all p < 0.005, multiple comparisons tests). These findings suggest that the conflict-related changes in 4 Hz coherence reflect changes in the VTA’s signalling to the ACC.

Because of the similarity of these PDC findings to the coherence findings, we were interested in whether VTA’s modulation of the ACC in the vertex would also show an “adaptation” effect by being elevated on the trial immediately following an error. Utilizing the same “relative to trial type” approach we used earlier (*vis a vis* Fig. 6), we assessed changes in vertex VTA → ACC 4 Hz PDC across during the three trials prior to the trials of interest as well as the two trials after trials of interest (Fig. 7b). First, we confirmed our linearized VTA → ACC PDC findings that indicate that vertex 4 Hz VTA → ACC PDC was maximal during incorrect choice trials, was minimal during instruction trials, and was normative during correct choice trials (trial 0 in Fig. 7b; F_2,998_ = 35.44, p < 0.0005; correct <incorrect, instruction <incorrect, instruction <correct, all p < 0.005, multiple comparisons tests). Next, we assessed the stability of the VTA → ACC PDC models during the three trials preceding an error, instruction trial, and correct choice. We detected no main effects of trial type, trial position, or their interaction in vertex VTA → ACC PDC in the three trials prior (−3 to −1 in Fig. 7b) to the trials of interest (0 in Fig. 7b). This indicates that VTA → ACC 4 Hz signalling was relatively stable prior to an error or instruction trial.

Given that the 4 Hz VTA → ACC PDC changes throughout the maze were similar to our earlier spectrographic analyses, we wondered whether 4 Hz VTA → ACC PDC would also exhibit an adaptaion effect. Specifically, we asked whether vertex 4 Hz VTA → ACC PDC was altered during the post-error trials (trial position 1 in Fig. 7b) with ANOVAs considering the changes in PDC by trial type across the trials of interest and the trials immediately afterwards (positions 0–2 in Fig. 7b). Similar to our ACC-VTA coherence results, we found a significant trial type x trial position interaction (F_4,6175_ = 13.56, p < 0.0005) but no significant main effects. Importantly, 4 Hz VTA → ACC PDC was greater during both error trials and the immediate post-error trial, as compared to correct choice and instruction trials (p < 0.005, multiple comparisons test). These results also indicate that the low 4 Hz VTA → ACC PDC that occurred during instruction trials increased in the following trial such that it was not significantly different from correct choice trials (p < 0.005, multiple comparisons test). There were no differences between the trial types two trials after the trials of interest (all p > 0.05, multiple comparisons test). These results indicate that VTA → ACC signalling was elevated as rats adapted their decisions following an error.

To check whether this adaptation effect was directly related to different types of decisions, we compared vertex VTA → ACC PDC during the trials immediately before and after trials of interest (i.e. trial positions −1 and 1 in Fig. 7b). Although we found no main effects of trial type or trial position, we found a significant trial type x trial position interaction (F_2,1996_ = 7.11, p < 0.0005). Post hoc multiple comparisons tests revealed that it was only during post-error trials that VTA → ACC PDC increased significantly (p < 0.005, multiple comparisons test). These results indicate that the VTA signalled to the ACC significantly more following and error than before one.

To complement our PDC results, we conducted amplitude cross-correlations^42^ of 3–5 Hz filtered ACC and VTA LFPs while rats were in the vertex of the maze. Because the rats’ movement speed, and thus time spent, in the vertex varied by trial type, we used a 1000 ms window for this analysis which began when the rats tripped the photobeam used to detect entry into the vertex region. This ensured that an equal amount of data was used for the cross-correlations. We found that as rats made incorrect decisions, the cross-correlations peaked at 30.77 ± 5.3 ms (p < 0.005, Kruskal-Wallis test), indicating that the VTA led the ACC. A similar maximum lag was detected during immediate post-error trials (mean ± SEM: 28.58 ± 6.4 ms, p < 0.005, Kruskal-Wallis test). In contrast, there was no indication of a significant lead/lag relationship during correct choices (mean ± SEM: 9.26 ± 3.5 ms, p > 0.05, Kruskal-Wallis test). These findings support our PDC models indicating that the VTA modulates the ACC during the commission of an incorrect choice as well as during the post-error adaptation.

### Post-error signals are unlikely to reflect a carry-over effect

Because we observed elevated VTA → ACC PDC in the vertex during both the error trial and the post-error trial, we wondered whether the latter signal was due to an error signal with a long time constant (e.g. a carry-over effect from the error trial) or was a unique and separately generated signal. To differentiate between these possibilities, we assessed how VTA → ACC PDC changed in the midstem region, immediately prior to vertex entry, for each trial type. ANOVAs and multiple comparisons tests returned no significant differences between trial types (all F < 1, all p > 0.1). This suggests that the elevated post-error signals detected in the vertex were unlikely to be the result of a carry-over effect from the prior error trial.

### Relationship to running speed

To rule out the possibility that our electrophysiological results were confounded by changes in the animals’ running speed, we conducted analyses of covariance (ANCOVA) with the residual normalised vertex VTA and ACC 4 Hz power and coherence as well as vertex 4 Hz VTA → ACC PDC as the dependent variables, respectively, with the corresponding residual running speed as a covariate, and the trial type as the independent variable. The resultant ANCOVAs indicated that after controlling for running speed, a significant proportion of variance was still explained by variations in trial type for all electrophysiological measures (all F_2,1199_ > 14, all p < 0.0005). These results indicate that our electrophysiological findings were not confounded by changes in the animals’ movement speed.

### Changes in instruction trials across experimental phases

**Figure 8:**
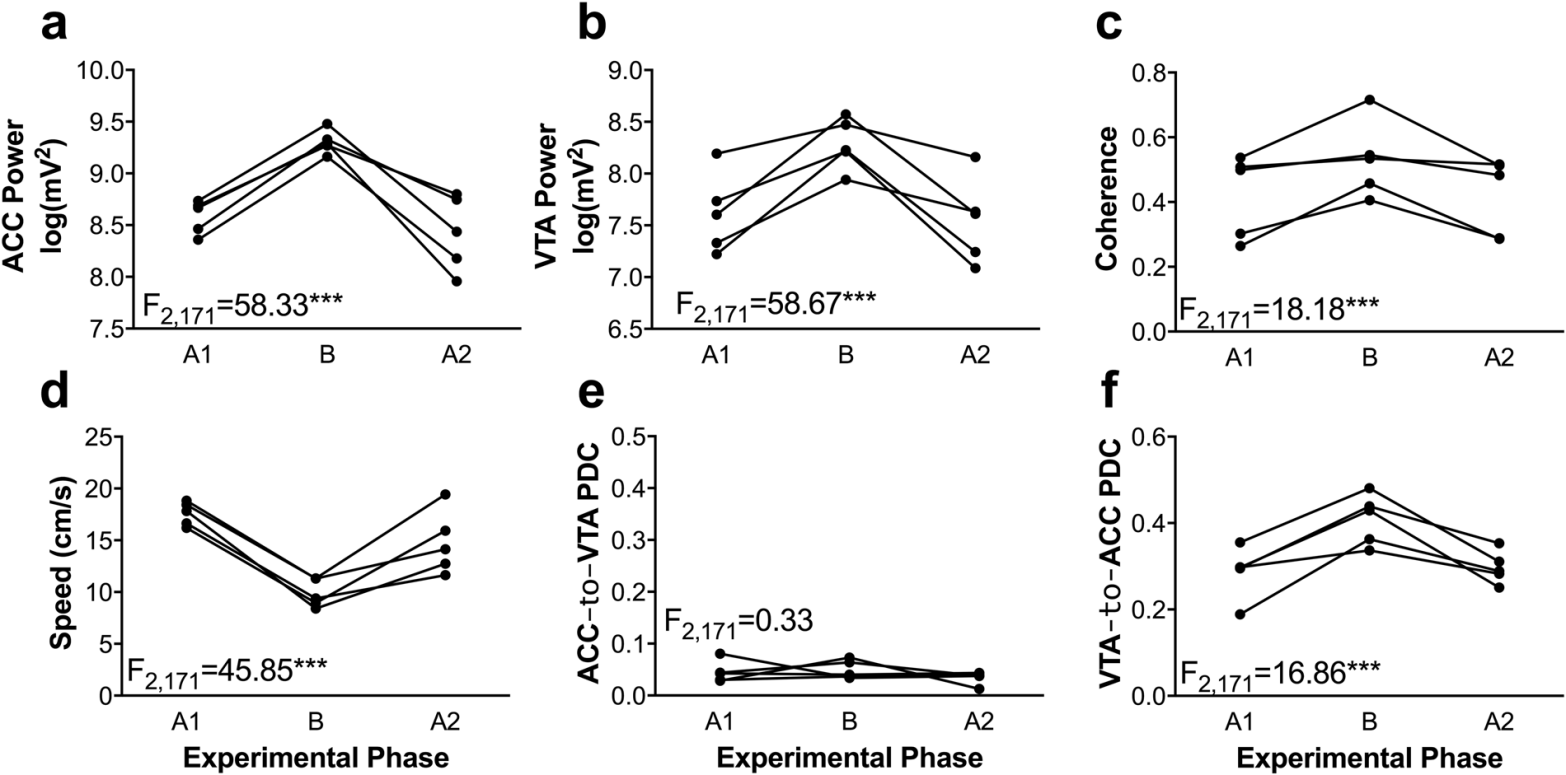
Experimental phase dependent changes vertex behaviour and electrophysiology during instruction trials. Here, we manipulated whether instruction trials forced animals towards the rewarded maze arm (phases A1 and A2) or towards the unrewarded, barrier-containing maze arm (phase B). Vertex ACC power (**a**), VTA power (**b**), ACC-VTA coherence (**c**), and VTA → ACC PDC (**f**) were greater during instruction trials which forced animals towards unrewarded, barrier-containing maze arms as compared to instruction trials which forced animals towards rewarded maze arms. Correspondingly, animals were slower to leave the vertex during instruction trials which forced animals towards unrewarded, barrier-containing maze arms as compared to their vertex running speeds during instruction trials which forced them towards rewarded, no-barrier maze arms (**d**). No differences in ACC → VTA PDC (**e**) were detected across the phases. Data were analysed according to the normalisation and regression parameters described for each dependent measure in the main text. Data are presented as the grand means of each animal such that each marker corresponds to the mean of one animal on the un-normalised (raw) scale of each dependent measure in one experimental phase. The lines connecting the markers indicate the change in the mean choices of individual animals.

Because some of the changes to ACC and VTA 4 Hz power, coherence, and PDC could be interpreted as resulting from variations in decision conflict, we asked whether we could systematically manipulate the conflict an animal experienced by altering the instruction trial. We hypothesised that when the rats’ prior experience was that instruction trials forced them towards rewarded trajectories, they would experience minimal conflict but that when instruction trials forced them towards unrewarded, barrier-containing trajectories, the animals would experience significant conflict. To test this hypothesis, the animals were subjected to three days of unrewarded, barrier-containing instruction trials (phase B in Fig. 8) before being run for three further days of rewarded, no-barrier instruction trials, identical to their initial training (phase A2 in Fig. 8).

The vertex-region results for correct and incorrect choice trials in phases B and A2 were nearly identical to those found in phase A1 (i.e. the results already reported). Therefore, we focused our analyses exclusively on the effect of instruction trial type on vertex-region electrophysiological and behavioural measures. ANOVAs considering the variation of 4 Hz ACC and VTA power, ACC-VTA coherence, VTA → ACC PDC, as well as running speed revealed a significant main effect of experimental phase (all F_2,171_ > 16, all p < 0.0005; see Fig. 8a–d,f). No effect of experimental phase was detected for vertex ACC → VTA PDC. These results indicate that when the instruction trials forced the animals towards effortful, unrewarded outcomes (phase B), the animals ran more slowly and that 4 Hz ACC and VTA power and coherence as well as 4 Hz VTA → ACC PDC increased during these potentially frustrating episodes, as compared to when they were directed towards a rewarded outcome.

## Discussion

We investigated ACC and VTA LFP power, coherence, PDC, and running speed as rats performed a reversal learning task where they decided whether to turn left or right in a maze such that only one choice option in any block of trials yielded reinforcement. PDC models of task-related directed relationships between brain areas revealed significantly elevated 4 Hz VTA → ACC communication as the animals made incorrect choices, while they were in the reward zone on such incorrect (unrewarded) trials, and during the implementation of the subsequent (correct) choice. This post-error signal had disappeared by the second trial after an error. Additionally, we found that ACC and VTA 4 Hz power and coherence were also elevated during error trials as well as during the subsequent post-error trial. In contrast, no measure of 4 Hz activity significantly varied during the three trials prior to either an incorrect or correct choice or during an instruction trial that directed the animal towards a correct choice. When instruction trials forced the animals to an unrewarded, barrier-containing arm, however, ACC and VTA 4 Hz power, coherence, and VTA → ACC PDC increased.

It is unlikely that our electrophysiological findings are confounded by changes in the animals’ movement speed as the two measures were dissociated under several conditions. For example, although the animals’ running speeds during phase A1 incorrect and instruction trials were comparable, ACC and VTA 4 Hz power, coherence, and VTA → ACC PDC were markedly different. Similarly, although post-error running speeds were comparable to those of post-correct-choice trials, ACC and VTA 4 Hz power, coherence, and VTA → ACC PDC were markedly different. Furthermore, after statistically controlling for speed as a covariate in an ANCOVA, the trial type remained a highly significant factor in the ACC and VTA. Therefore, rather than being a secondary consequence of behavioural change, our electrophysiological data appear to represent fundamental differences in underlying neural activity.

The differences in running speed and ACC activation that we observed prior to an incorrect choice can be compared to the numerous studies demonstrating that greater choice reaction times and ACC activation correspond to greater choice conflict^12,33,34^. Thus, our results suggest that VTA-ACC signalling may be a component in choice conflict. The increase in VTA → ACC signalling that was associated with this response may have had several functions, including sharpening attention, increasing signal-to-noise in memory recall, and generating (or resulting from) an anxiety response. These are not mutually exclusive outcomes. In the instruction trials, animals may have been sanguine about being forced to take a rewarded path (phases A1 and A2) whereas they may have been frustrated and unwilling to follow an unrewarded, barrier-containing path (phase B). There was an increase in VTA → ACC signalling during the ‘negative’ instruction trials (phase B), however, in this case, memory recall and attention is unlikely to be activated over and above the ‘positive’ instruction trial protocol, suggesting that the signal may be related to frustration, anxiety, or conflict.

In light of evidence indicating a central role for the VTA in the anticipation and experience of feedback^43–46^, it is possible that the elevated VTA → ACC communication we observed while the rats were in the reward zone during incorrect choices may be a means by which such feedback is made available to the ACC, thereby tuning cortical representations in order to adapt future behaviour. This is plausible considering that the rats adapted their behaviour on the subsequent trial.

We also found that VTA → ACC communication increased as the rats made the post-error (correct) choice. One interpretation suggested by stable-coding theories of working memory^29,47,48^ is that this increased communication could be a carry-over from the activation that occurred on the error trial (i.e. an error signal with a long time-constant). We tested this possibility by comparing post-error and post-correct-choice VTA → ACC PDC in the midstem because if this signal did reflect a carry-over effect, post-error VTA → ACC PDC in the midstem should be greater than the post-correct activation. This was not the case, however, and, in fact, VTA → ACC PDC on error trials decreased in the barrier region, immediately after the error response. Another possibility, suggested by dynamic-coding theories of working memory^49–51^, is that the error signal persists across a variety of neuronal timescales (e.g. neurons with different firing time-constants^49,51^). According to this view, our spectral measures would not have detected such a temporally distributed representation. Because our post-error midstem results do not support a stable coding interpretation, future studies utilizing high-density recording techniques will be crucial to characterize whether and how error signals dynamically persist in working memory across several timescales^52^.

An alternative possibility is that increased 4 Hz VTA → ACC PDC on the post-error trial reflects post-error adaption. This interpretation is consistent with the predictions of computational models^16,17^ and the results of a recent study^19^ of how the VTA influences cortical regions. For example, Ellwood *et al*.^19^ found that tonic and phasic stimulation of cortical VTA DAergic terminals differentially caused mice to persist or deviate from their prior behaviour. Additionally, numerous studies suggest a central role for cortical DAergic activity in behavioural flexibility^21,22,53,54^. Thus, one interpretation of these findings is that VTA bottom-up projections to the ACC underlie a circuit via which cortical representations can integrate recent feedback and thereby influence future decision behaviour, perhaps by altering the coding properties of cortical neurons^24^.

It is also noteworthy that these present findings differ from our prior study of the ACC-VTA circuit^28^. In our prior study, we found that the unexpected absence of a barrier increased ACC-VTA coherence but that this occurred in a top-down, ACC → VTA direction. Although we did detect an increase in ACC-VTA coherence associated with the absence of the barrier in this experiment, we did not find top-down directionality. We speculate that this difference relates to the natures of the tasks and what the barrier, or its absence, signifies in each task. For example, a major difference between the current and prior study is that in our prior study there was no choice component such that the presence or absence of the barrier was surprising for the rats. Therefore, perhaps, the absence of the barrier may have simply led the rats to anticipate the reward earlier than otherwise, with the absence of additional effort signalling that the reward was unexpectedly “closer.” In contrast, in the present experiment, the contingencies associated with each maze arm during each block were static; thus, the absence of the barrier during correct choices may have not been surprising for the rats although its absence was similarly a cue that the reward was “closer” (although both barrier and non-barrier trials were rewarded equally). In line with this anticipation interpretation, the presence of the barrier in this experiment was associated with no reward and may have indicated to the rat that the reward was “further” perhaps reducing the animals’ anticipation of that reward. When considering the findings of both experiments, the notion of anticipation can account for the dynamics of ACC-VTA 4 Hz coherence, where the onset of the ACC → VTA directionality may be modulated by the probabilities associated with response requirements (e.g. the probability of a barrier being present or number of lever presses required) and/or reward (e.g. availability and magnitude). Future studies utilizing a behavioural paradigm which provides the animals certain and dynamically-uncertain decision options in terms of response requirements, reward availability, and reward magnitude are required to resolve these questions.

When considering our prior^28^ and current findings through the lens of task-model theories of ACC function^4,13,55^, the data suggest that the ACC and VTA regions do not generally encode or respond to effort itself but rather appear to be driven by what the effort component of a task means in context. Thus, our prior findings suggest that ACC → VTA activity may reflect the role of the circuit in anticipation. In the current task, with ‘positive’ instruction trials, a simple ‘repeat previous trajectory’ strategy maximises reward acquisition while minimising cognitive demand. When an error occurs, however, this strategy must be supressed on the following trial, and a different feedback-driven response must be implemented. The VTA → ACC signalling that occurs during post-error adaptation may, therefore, represent a mechanism by which an animal integrates and utilises feedback, perhaps through an enhancement of signal to noise in the underlying neuronal task representations^10,13,46,56^.

In conjunction with Ellwood *et al*.’s^19^ and Tervo *et al*.’s^4^ results, our data suggests that low-frequency VTA → ACC signals might modify the composition of ACC neuronal task models such that this signal could induce, respond to, and/or correct for decision conflict. A causal role for VTA → ACC signalling in post-error feedback utilisation could be directly tested in a similar reversal decision paradigm with the predictions that photoinhibition of VTA DA terminals in the ACC as rats make their decision during the immediate post-error trial would decrease 4 Hz coherence and also decrease the likelihood that that decision would be correct. Such a finding would confirm the role of mesocortical signalling in the ACC’s integration and utilization of feedback to adapt decision making. Similarly, photostimulation of intra-ACC VTA DA terminals during a default decision might help to determine whether the role of VTA → ACC signalling is causal of, or responsive to, decision-conflict.

It is important to note several caveats when interpreting our results. First, although we immunohistochemically verified that our VTA electrodes were located within a region containing DA neurons, it is unlikely that our VTA → ACC PDC models captured purely DAergic signalling. Rather, they likely reflect the aggregate of directed information transfer between the structures (e.g. co-localized glutamatergic and GABAergic neurotransmission^57,58^). Thus, future experiments could use fibre photometric measures of VTA DAergic activity in the ACC to determine the degree to which our PDC results correspond to changes in mesocortical DA signalling. In a similar vein it is possible that the LFP signals we observed in the VTA and the ACC result from volume conduction from nearby structures^59^. Although we cannot rule out this possibility, the changes in coherence and PDC between the two structures, indicates at least, a degree of independence between the signal generators. Furthermore, in a separate experiment (data not shown) run with the same animals and apparatus, we also recorded single units from ACC. Unit firing was seen to phase lock to the local ACC LFP, suggesting that this LFP signal was locally generated. Some ACC cells also phase locked independently to the signal on the VTA, but not the ACC, electrode, consistent with the proposal that ACC units were influenced by a signal originating at or around the VTA.

An additional caveat is that our task design did not allow us to isolate the source of the rats’ putative conflict. It is possible, therefore, that their behaviour was influenced by uncertainty about which option was currently optimal. Future studies utilising a dynamic probabilistic reversal design^60,61^ where the probabilities associated with response options both reversed and changed could be used to characterize the ACC-VTA circuit’s response to uncertainty and as the circuit’s role in other forms of task-model updating^13^.

In conclusion, our data integrate and extend the evidence indicating the ACC’s role in decision conflict and adaptation and suggest that VTA → ACC signalling is important for these processes. Together, our results support the idea that feedback alters cortical neuronal representations via changes in mesocortical signalling and that this same mechanism is also associated with response conflict. Future work will expand mechanistic insight into this phenomenon by examining how changes in VTA → ACC signalling correspond to changes in the neuronal representations thought to facilitate behavioural flexibility.

## Materials and Methods

### Subjects and animal use statement

All experimental procedures have been reviewed and approved by the University of Otago Animal Welfare Office and all methods were performed in accordance with the relevant guidelines and regulations. Five male Sprague Dawley rats (Hercus-Taieri Resource Unit) between six and eight months of age, weighing 400–550 grams were used in this study. Rats were single housed in translucent plastic cages containing pine chips and maintained on a 12 hour light/dark cycle. All training and experimentation occurred during the light phase. After two weeks of daily handling and weighing during which rats had *ad libitum* access to food, rats were food deprived of standard rat chow (Speciality Feeds) to no less than 85% of their free-feeding weight to promote interest in reward during the experiment. Water was available *ad libitum* at all times in the home cage. These rats were also used to collect a dataset which was different from the present study.

### Preoperative training

During the initial week of training, rats were individually habituated for 15 min/day to the experimental apparatus, a figure-of-eight shaped runway which contained one touchscreen, seven photobeams, ten pneumatic rams (to open and close doors in the maze), two climbable 30 cm barriers mounted on servo motors, and two peristaltic pumps (to dispense condensed milk in the reward regions). The apparatus was controlled by a network of five Arduino microcontrollers (Arduino LLC, Somerville, MA, USA; see Fig. 1a). During habituation, condensed milk was freely available from plastic wells located in the reward zones. In the second week of training, rats were trained to run in a unidirectional manner. This was achieved by forcing the rats to turn either left or right in the vertex and delivering 0.5 ml of condensed milk for each completed circuit. Rats were prevented from reversing course through the maze by photobeam-controlled doors. The rats were not paused between trials and set their own pace. This phase typically took between five and eight training sessions.

Once rats were running unidirectionally, they were trained to initiate trials by pressing a wall-mounted touchscreen. The touchscreen indicated a successful press by turning from black to bright red and the release of an adjacent start gate. There was no delay between screen pressing and the lowering of the start gate. Touch-press shaping took between one and four 15 minute training sessions and training was considered complete when rats completed at least 30 trials in a 15 minute training session. In total, this phase took approximately six 15 minute training sessions.

The next training phase required rats to climb over a retractable, servo-mounted 30 cm barrier on route to the reward zone. The servo motors both inserted and removed the barriers according to the programmed task protocol carried out by the Arduinos. These barrier-training sessions were to habituate the rats to the presence of the barriers and for them to learn how to climb over them. 0.2 ml of condensed milk was delivered at the completion of each trial. Barrier training was considered complete when rats completed at least 30 trials for three consecutive 15 minute sessions. This typically took four training sessions.

Following this initial training procedure but before surgery, the animals were trained in a cost-benefit decision task similar to the one used by Hillman & Bilkey (2010) which was used to gather data that was not used in the present study.

### Surgery

Once all rats met the training criteria, they were anaesthetized under isoflurane and stereotaxically implanted in the ACC (AP: 2.7 mm, ML: 0.4 mm, DV: −1.8 mm from dura) with seven 25 μm Formvar-coated nichrome wires (one tetrode and one tritode; California Fine Wire) mounted on a 3D-printed adjustable microdrive assembly and the VTA (AP: −5.3 mm, ML: 1.0 mm, DV: −8.2 mm from dura) with a one non-moveable 127-μm-diameter, nickel chromium-coated wire. The electrodes were grounded by soldering a wire to a jeweller’s screw implanted in the cerebellum. The assembly was fasted to the skull with jeweller’s screws and acrylic dental cement. Following the surgery, animals were allowed 10 days to recover, during which time they had *ad libitum* food and water. After 10 days, rats’ food was reduced to maintain the animal at ~85% of their free-feeding weight to optimize behaviour during the experiment.

### Postoperative training and reversal task

After 10 days of recovery, the rats were reintroduced to the maze with head plugs connected to a tethered head state that housed three light emitting diodes (LEDs) for tracking. The rats then underwent the cost-benefit training and task, which was not a part of the present study. Following completion of the cost-benefit analysis experiment, the rats were retrained for the present experiment. Our spatial decision reversal task required rats to turn left or right in a continuous T-maze such that in any block of approximately 12 trials only one choice option yielded reinforcement (see Fig. 1b). Block lengths were on a variable-length schedule such that each block consisted of 12 ± 2 trials. Each block consisted of three different trial types: one forced ‘instruction’ trial, a variable number of free choice trials (10 ± 2), and one working memory trial. Each block began with an ‘instruction’ trial in which rats were forced to follow the reinforced path by blocking access to the non-reinforced maze arm. Following an instruction trial, rats were given 10 ± 2 free-choice trials. Rats initiated a trial by pressing a wall-mounted touch-screen which opened a start gate. Upon reaching the vertex maze region, rats had to decide whether to turn left or right based on recent choice-outcomes. Correct decisions were reinforced with 0.2 ml of condensed milk which was diluted with tap water at a 1:3 ratio (Highlander, Nestle New Zealand, Auckland, New Zealand); incorrect decisions were punished by inserting a climbable 30 cm barrier into the path of the rat as well as the omission of reward. Although a barrier was only encountered following an “incorrect” choice, we refer to the maze arms as the “barrier region” in the following figures to avoid using different terms for different trial types to describe the same maze region. In order to prevent the rats from reversing course and returning to the maze arm, a photobeam-controlled gate closed as the rats approached the reward well. We trained the rats to run all the way through the reward zone to the far reward well so as not to catapult the animals out of the apparatus when the photobeam-triggered gates were raised at the completion of each trial. The rats were not paused between trials and set their own pace, running in a continuous, uninterrupted manner.

Pseudorandomly interleaved in the sequence of choice trials were working memory trials such that one working memory trial occurred per block. A working memory trial began similarly to a free-choice trial but, upon reaching the vertex, all maze doors were closed for five seconds so that the rat was paused in the midstem and vertex regions of the maze. Following the five-second pause, the left and right vertex doors opened, which then allowed the rats to carry out their decisions. Working memory outcome contingencies were identical to free-choice outcomes. However, to retain focus in this paper and because our results for these working memory trials were nearly identical to prior reports^29^, we omitted them from the subsequent analyses. After approximately 12 ± 2 trials, a new block began where the reinforced rule was reversed and rats were ‘instructed’ regarding the new rule in a forced-choice trial. In total, rats completed six blocks with a total of approximately 80 trials per data collection session.

This training was conducted as the task protocol itself for 10 days where the training criteria was for the animal to make 80% correct choices in a given session. Following this 10-day period, they were run in a variant of the task where the ‘instruction’ trial at the start of each was altered, forcing the animal to go to the incorrect side where they would have to climb a barrier and get no reward. The data acquisition protocol consisted of a three-stage ‘ABA’ design which took a total of 9 days (3 days per phase). A given day’s recording procedure involved first recording the rats roaming a familiar open field for 10 minutes before the rats were then transferred to the maze where completed 80 trials per day. The type of instruction trial was varied across the phases such that the first three days (phase A1) had rewarded instruction trials, the fourth through sixth days had unrewarded, barrier-containing instruction trials (phase B), and the seventh through ninths days (phase A2) had rewarded instruction trials and was identical to phase A1. All rats experienced all phases identically.

### Electrophysiology and tracking

ACC and VTA local field potential (LFP) activities were monitored via the DacqUSB acquisition system (Axona, Ltd., St. Albans, UK). ACC LFPs were recorded from a single wire contained in a tetrode and VTA LFPs were recorded from a single nickel-chromium wire. Acquired LFPs were low-pass filtered at 500 Hz and sampled at 4800 Hz. The rats’ movement through the maze was recorded by a ceiling-mounted video camera which tracked the LEDs mounted on the head stage. Tracking data was sampled at 50 Hz and was made available to the dacqUSB system. The dacqUSB system also recorded key task events (e.g. touchscreen presses, beam breaks) via digital inputs from the Arduino network. Subsequent analyses were conducted with MATLAB R2016b (The Mathworks, Boston, MA, USA).

### Local field potential power and coherence

Time-resolved LFP power and coherence were calculated via multi-taper spectrograms and coherograms (Mitra & Bokil, 2008) which used three tapers, a one-second reading window, and 85% overlap amongst windows.

### Partial directed coherence for detecting signal directionality

The directionality of task-related relationships between the ACC and VTA LFPs were assessed with a partial directed coherence (PDC) algorithm, which uses multivariate autoregressive modelling to exploit the predictability of information in one brain area by past activity in another^27^. One advantage of PDC over other measures of directionality, such as amplitude cross-correlations (e.g. Adhikari, Sigurdsson, Topiwala, & Gordon, 2010), is that PDC allows for testing the possibility of simultaneous bi-directional communication, which is particularly likely to occur between two reciprocally connected areas, such as the ACC and VTA^57,62^.

Our implementation of PDC followed Boykin, Khargonekar, Carney, Ogle, & Talathi’s^26^ method. Briefly, we linearly detrended the unfiltered LFPs and then fitted relevant sections of the ACC and VTA LFPs to an autoregressive model where the maximum model order was determined according to Akaike’s^63^ information criteria. We did this through the ARfit MATLAB package^64^ which yields a set of time-resolved coefficient matrices. Those coefficient matrices were then frequency-resolved via the Fourier transform. From here, we followed Baccala and Sameshima’s^27^ mathematical definition of PDC for each frequency component.

### Regression to control for between-subject variance

We used a regression-based method, first reported by Ma, Hyman, Lindsay, Phillips, & Seamans^32^, to control for between-subject variance. This allowed us to pool/analyse via multifactorial ANOVAs (*anovan* in MATLAB) the individual trials across all 5 rats.

The general approach we used was first to normalize the data via the feature scaling formula^39^, which rescaled the data between 0 and 1, and to then fit a regression model (*regress* in MATLAB) where a dependent measure of interest was regressed against a subject factor (i.e. a factor indicating which animal a particular data point was obtained from). The resulting model captured the variance in the dependent measure of interest that was explained by differences between the rats. The residuals, however, contained data that reflected the underlying variance in the parameter, independent of the effect of subject. It was these residuals which we analysed with the multifactorial ANOVAs. The benefit of this approach was that it more clearly showed the same results as rat-by-rat within-subject analyses and also revealed new trends. The other advantage of this approach was that it made both the interpretation and the reporting of the results more clear and concise.

### Histology and electrode placement verification

Once the study was completed, rats were deeply anaesthetized with isoflurane and recording sites were electrolytically lesioned with direct current (2 mA for 2 seconds) before transcardial perfusion. Microlesions marking the electrode placements were identified in 50 μm sections of Nissl stained, formalin-fixed tissue. VTA electrode placements were verified via sections immunohistochemically labelled for tyrosine hydroxylase (TH; anti-TH primary antibody was AB152 from Millipore). TH is a marker of dopamine producing neurons and has previously been used to define the boundary of the VTA^65^. All VTA electrode placements were within the TH+ area, indicating that we recorded LFPs originating from the location of dopaminergic neurons (see Fig. S2).

### Data availability

The datasets generated during and/or analysed during the current study are available from the corresponding author on reasonable request.

## Electronic supplementary material

Supplementary Information
