# Supplementary material for: Conflict and adaptation signals in the anterior cingulate cortex and ventral tegmental area
Source: Sci Rep. 2018 Aug 6;8:11732. doi: 10.1038/s41598-018-30203-4 (PMC6079061; doi:10.1038/s41598-018-30203-4)
Supplement: Supplementary file 1 — Supplementary Information [file 41598_2018_30203_MOESM1_ESM.docx]

Scientific Reports

Supplemental Information

Conflict and adaptation signals in the anterior cingulate cortex and ventral tegmental area

Thomas W Elston, Shivam Kalhan, & David K Bilkey


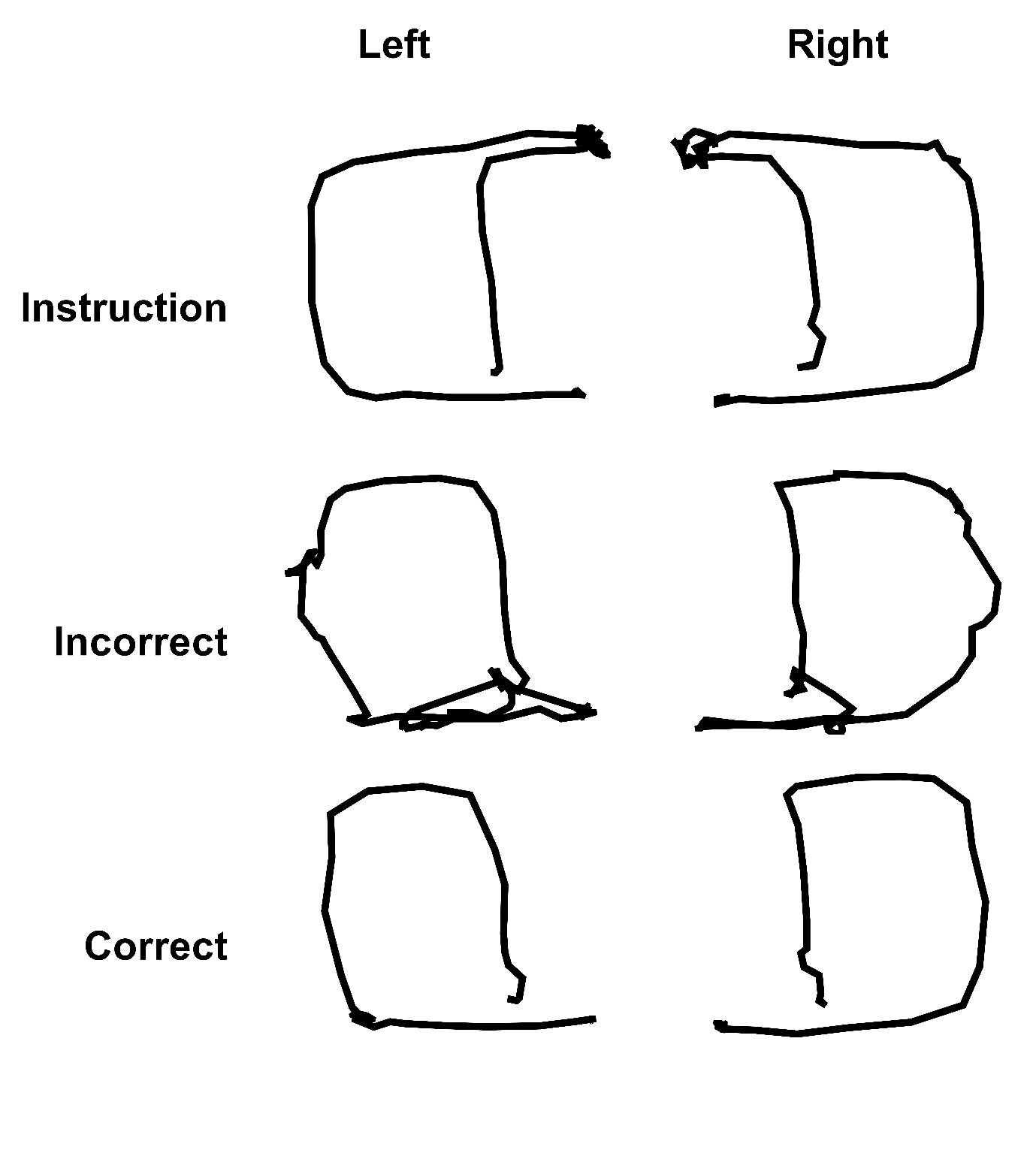


Figure S1. Example single-trial movement trajectories by trial type and maze side. These data are all from the same recording session of rat 4 during phase A1.


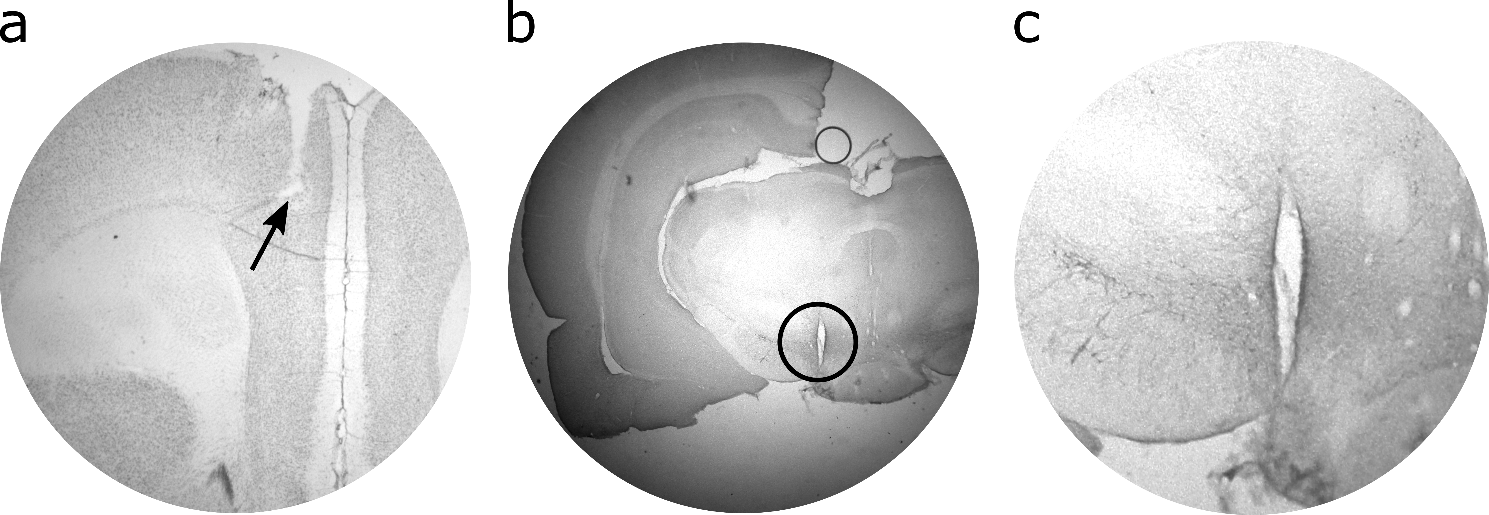


Figure S2. Histology indicating electrode placements in the ACC and VTA. **(a)** Nissl-stained section where the arrow indicates the tip of the electrode bundle targeted at the ACC. **(b)** A VTA section that was immunohistochemically stained for tyrosine hydroxylase (TH), a marker of dopamine-producing neurons. The circled area highlights an electrolytic lesion in the VTA. **(c)** Enlargement of the circled area in (b) showing that the VTA-targeted area was within the TH+ area.


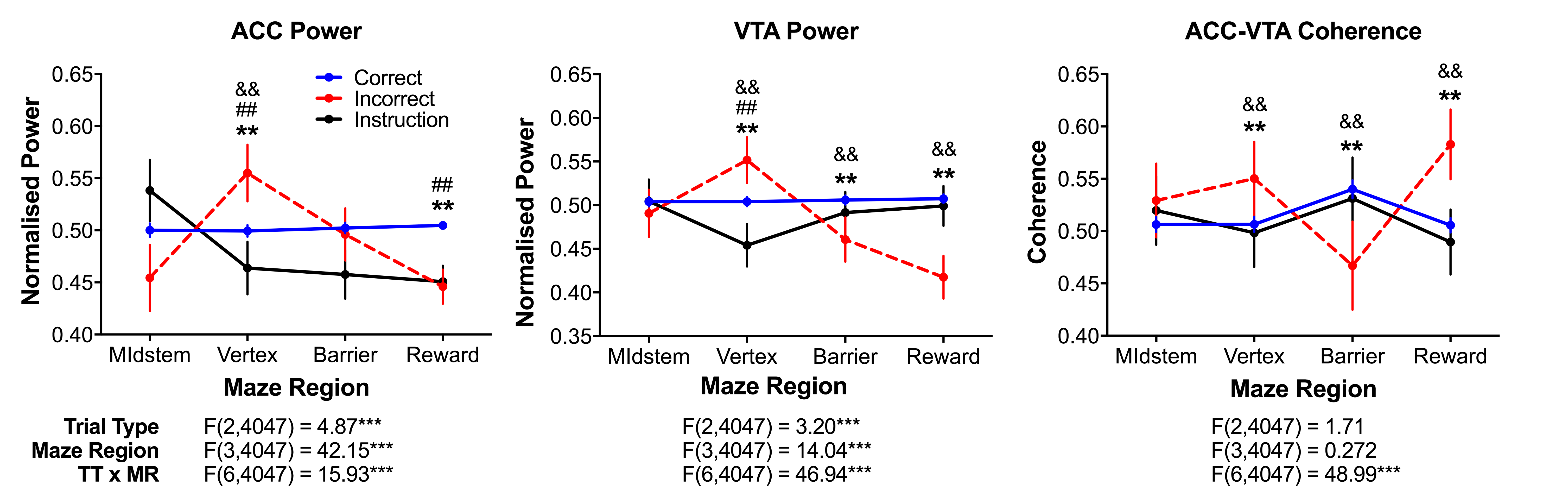


Figure S3. Linearized ACC and VTA 4Hz power and coherence without regressing off between-subject variance. These data relate to Figure 5 in the main manuscript. ** indicates that correct choices were significantly different from incorrect choices at the p < .005 significance level via multiple comparisons tests; && indicates that incorrect and instruction trials were significantly different at the p < .005 significance level via multiple comparisons tests; ## indicates that correct choices and instruction trials were significantly different at the p < .005 significance level via multiple comparisons tests.


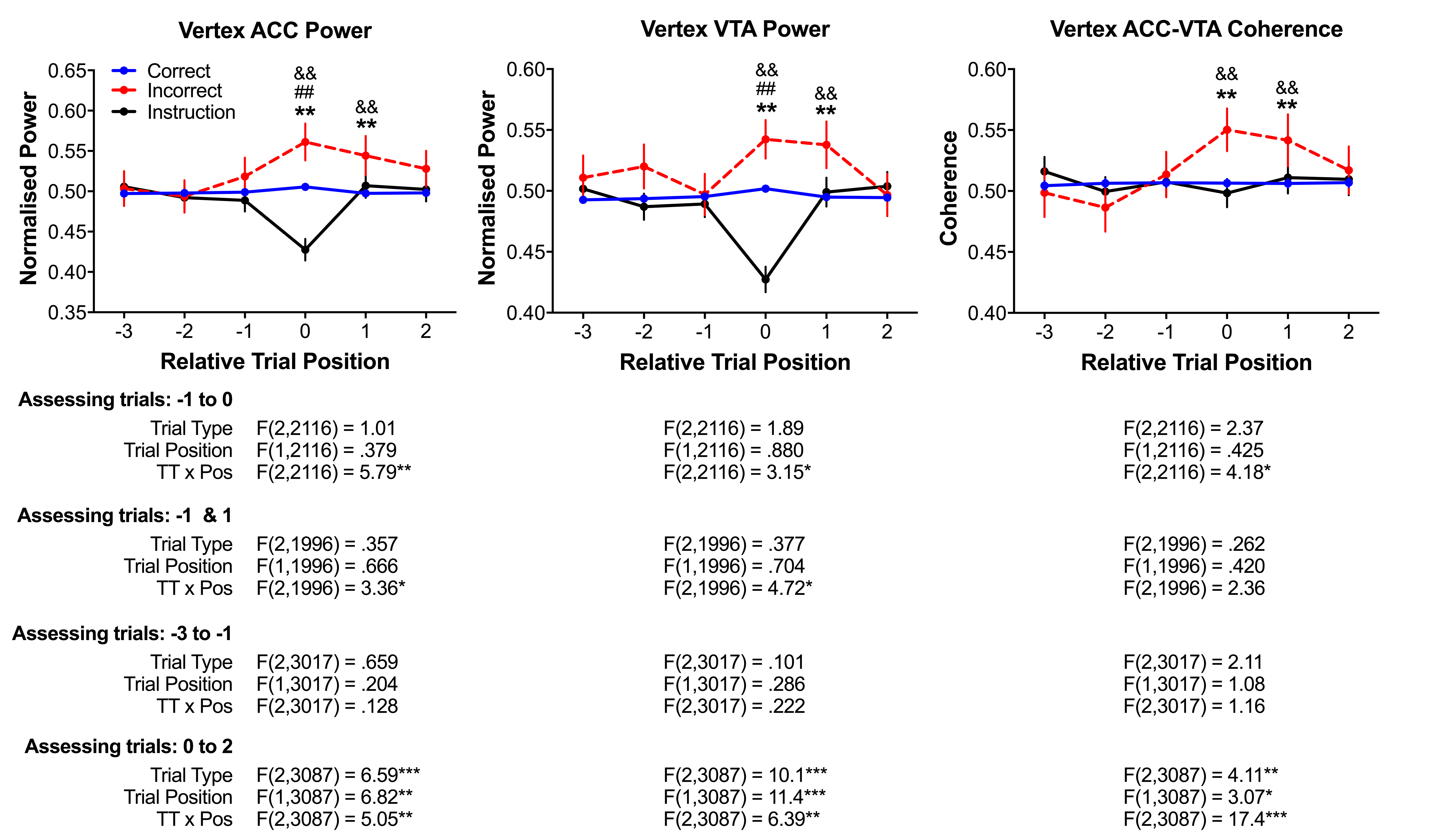


Figure S4. Changes relative to a trial-type of interest. These data have not been processed with regression procedure used throughout the main text. These data relate to Figure 6 in the main manuscript. ** indicates that correct choices were significantly different from incorrect choices at the p < .005 significance level via multiple comparisons tests; && indicates that incorrect and instruction trials were significantly different at the p < .005 significance level via multiple comparisons tests; ## indicates that correct choices and instruction trials were significantly different at the p < .005 significance level via multiple comparisons tests.


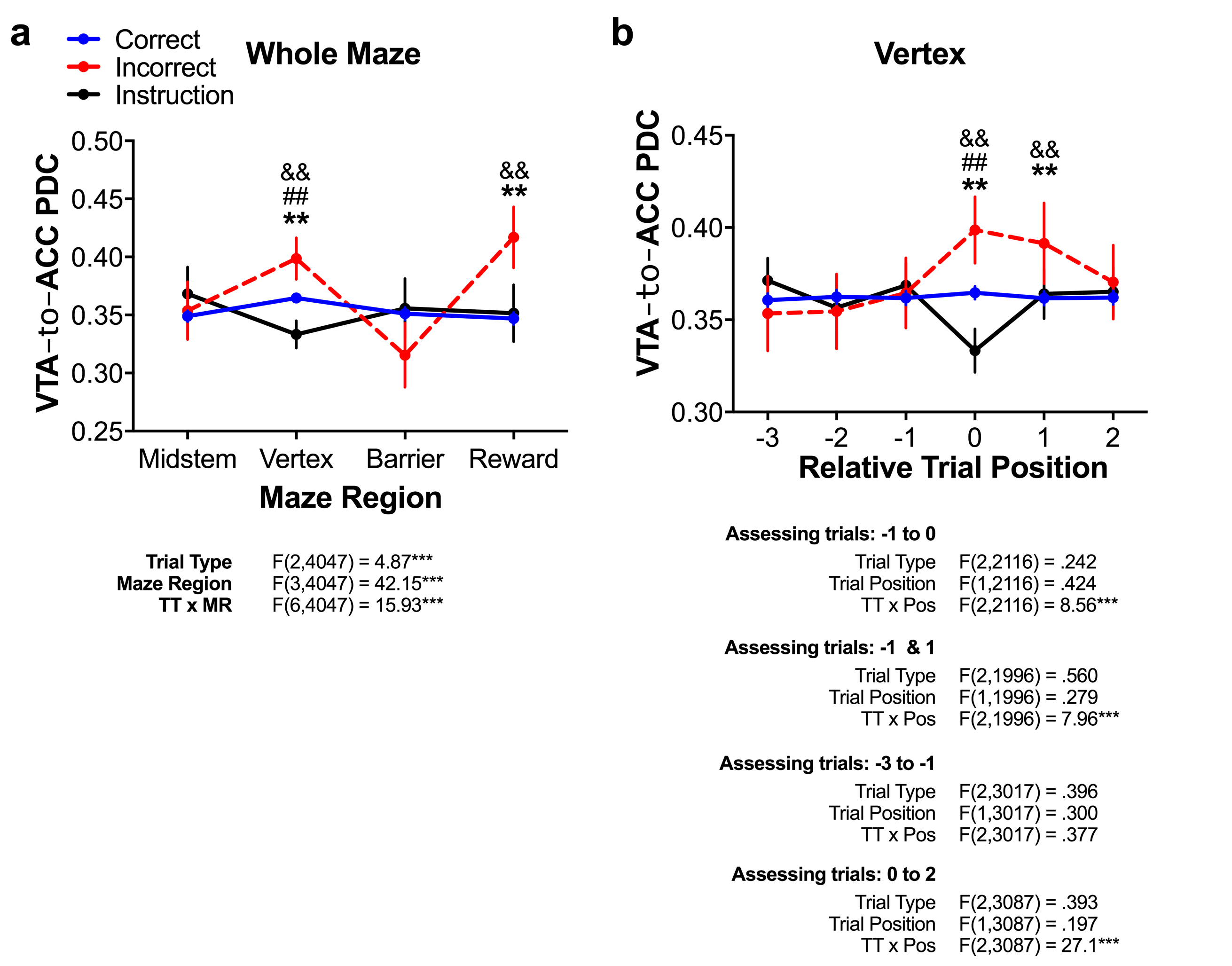


Figure S5. 4Hz VTA🡪ACC PDC models by trial-type across the maze regions (a) and relative to a specific trial-type of interest (b). These data have not been processed with the regression procedure used throughout the main manuscript. These data relate to Figure 7 in the main manuscript. ** indicates that correct choices were significantly different from incorrect choices at the p < .005 significance level via multiple comparisons tests; && indicates that incorrect and instruction trials were significantly different at the p < .005 significance level via multiple comparisons tests; ## indicates that correct choices and instruction trials were significantly different at the p < .005 significance level via multiple comparisons tests.
